# Event and Comment

**Published:** 1916-05

**Authors:** 


// DENTAL REGISTER

Vol. LXX MAY, 1916 No. 5

### EVENT AND COMMENT

A NEW METHOD OF SHOWING CLINICS TO LARGE CONVENTIONS. The annual meeting of the Michigan State Dental Society was held in Detroit, April 13 to 15, and it was probably more largely attended than any other meeting of this Society. The essays and addresses were most interesting and of great value. The feature of the program that attracted the most attention and was in itself unique was the clinical program. A new method of giving clinics was tried out and in some respects has much to commend it. The plan was to engage from eight to ten clinicians in the demonstration of a certain subject, and have a certain number of persons view each part of the clinic for a given time and then pass on to the next demonstration. For instance, the crown and bridge section was given in seven distinct parts, or steps, and each step was demonstrated by a different operator. The first operator demonstrated two kinds of hand carved porcelain crowns; the next, plate and dowerl crowns; the next, cast cusp and seamless gold crowns; the next, Richmond crowns; the next, setting and investing bridge parts; the next, completed fixed bridges; the next, inlay attachments and removable bridges.

The same method was used in showing methods of making, from impressions and models, inlays, crowns and bridge attachments; also for various steps in plate prosthesis, porcelain crown work, prophylaxis and pyorrhea, and for pathology and surgery. The work was devised and executed by the "Detroit Clinic Club," and was carried out with admirable system and detail. The principle objection to the plan seems to have been that it was practically impossible for any visitor to see more than one subject each session, and as there were but two clinical sessions in the entire meeting, no one could see more than two subjects; consequently the plan will have to be used for three years at least to give every one a chance to see all the subjects. Another objection seemed to be that as only fifteen minutes were allowed to see each step, each demonstrator was greatly hurried in presenting his part, and as some persons were better prepared and some were better talkers, there naturally was more or less unsatisfactory work done. The method is valuable, in our judgment, because it enables a large number of young men to take a part in the clinics, as they will not be embarrassed by a crowd, and each one will always have an audience that is bound to remain in a silent and attentive attitude while the demonstrator does his stunt, and as the demonstrator knows how much time he will have he can prepare beforehand his work accordingly. We don't believe the method will ever become popular as it does not permit one to see the clinics in which he is particularly interested, without spending much time on things for which he has no special use, and also because it does not permit the clinician to present other collateral arguments in support of the merits of his particular methods. The method, however, is interesting as evidence of the fact that there is a great interest, not alone in technical procedures, but in the fact that we are inventing better methods of disseminating technical ideas in our professional societies. The clinical idea will always be an interesting feature of convention work, but it is the most difficult part of the convention program to present satisfactorily. The old plan of the individual clinic will always have a place, and the section methods no doubt will meet with much favor, but we believe that better results will ultimately come from some method where the trained clinician can interest a larger audience by some form of clinic that can be seen at considerable distance, by means of stereoscopic projections, or by models and drawings that make an appeal through the eye whilst the operator reaches the intellect in a clear and carefully prepared word picture. This means better prepared clinicians or demonstrators, and might, of course, confine our clinics to a comparatively smaller number of operators than we frequently find on the programs of our larger societies.

ANNUAL REPORT OF THE FORSYTHE DENTAL INFIRMARY. The first report of this institution shows some interesting features as to what has been accomplished and offers suggestions of future usefulness. Nearly 20,000 patients were treated during the year, and an average of 413 patients were seen each day, of which 356 were strictly dental patients. To care for such a large number of patients 24 full-time operators were in daily attendance, and an average of 28 half-time operators, besides 140 on the visiting staff. 74,000 fillings were made, 38,000 treatments and 16,000 prophylaxis cases were treated; 84,259 visits were recorded, with a grand total of 128,404 operations. There were 2,222 nose and throat examinations, with 480 operations. In the Orthodontic Clinic 111 patients were treated with 2,868 treatments. Over 16,000 teeth were extracted, 5,545 of which were first permanent molars. Three cases of cleft palate were treated, and three cases of fractured jaws were treated. Besides this, 586 lectures on Oral Hygiene were given. Certainly this plant has been busy, and its future influence for good teeth will be tremendous.

